# A formal analysis method for composition protocol based on model checking

**DOI:** 10.1038/s41598-022-12448-2

**Published:** 2022-05-19

**Authors:** Meihua Xiao, Hanyu Zhao, Ke Yang, Ri Ouyang, Weiwei Song

**Affiliations:** School of Software, East China Jiaotong University, Nanchang, 330013 China

**Keywords:** Computer science, Information technology

## Abstract

Protocol security in a composition protocol environment has always been an open problem in the field of formal analysis and verification of security protocols. As a well-known tool to analyze and verify the logical consistency of concurrent systems, SPIN (Simple Promela Interpreter) has been widely used in the analysis and verification of the security of a single protocol. There is no special research on the verification of protocol security in a composition protocol environment. To solve this problem, firstly, a formal analysis method for composition protocol based on SPIN is proposed, and a formal description of protocol operation semantics is given. Then the attacker model is formalized, and a message specification method based on field detection and component recognition is presented to alleviate the state explosion problem. Finally, the NSB protocol and the NSL protocol are used as examples for compositional analysis. It is demonstrated that the proposed method can effectively verify the security of the protocol in a composition protocol environment and enhance the efficiency of composition protocol verification.

## Introduction

With the continuous development of network technologies such as the Internet of Things (IoT) and cloud technologies, the network has brought great convenience to people’s lives, but there are also many hidden security risks^1,2^. All aspects of network security such as malware detection^3^ and web attack detection^4^ are getting more and more attention. The security protocol is a high-interoperability protocol based on a cryptographic system. It is one of the important means to solve network problems too. How to improve the reliability of the security protocols is a current research focus. Compared with informal methods difficult to guarantee the protocol security, formal methods^5^ based on mathematics can find the vulnerabilities of security protocols, which are not easily found with other methods. In recent years, there have been many successful formal analysis methods and tools to analyze security protocols^6,7^, however these methods are mostly limited to a single protocol environment and cannot analyze the security of a protocol in a composition protocol environment. Actually, the real-life applications of security protocols are often very complicated, and multiple different protocols may be running on the same communication network, so the detection of composition protocol attacks cannot be ignored.

Multi-protocol attack was first proposed by Kelsey^8^, and then Meadows^9^ pointed out its importance in future security protocol analysis. Model checking^10^ is one of the important methods for formal analysis of security protocols. Formally, assuming that a system is S, and the desired system property is a logical formula $\varphi$ , then model checking is to verify whether the system S satisfies $\varphi$ , that is, whether $\mathrm{{S}} \models \varphi$ holds. It is highly regarded because of its high degree of automation and intuitive response to the checking results and giving counterexamples when the property is violated. Panti et al. applied the model checking method to detect composition protocol attack for the first time and proposed a method to automatically verify the security of the composition protocol system on the model checking tool NuSMV^11^, though the efficiency is slightly lower. Cremers et al. proposed a formal verification method for security protocols in a composition protocol environment and developed the model checking tool Scyther to realize the automatic confirmation of composition protocol attacks^12^, but their work is limited to cases where the number of protocols is small. In addition, there are many researchers^13–17^ dedicated to composition protocol attack checking.

SPIN^18,19^ is a model checking tool developed by Holzmann^20^ to analyze the logical consistency of concurrent systems. It has a good algorithm design, excellent checking capabilities and high degree of automation. Over the past two decades, SPIN has been widely used in the analysis and verification of the security of a single protocol. In 2002, Maggi proposed a method to statically analyze the intruder’s knowledge set^21^ and applied SPIN to analyze the NS protocol. Although the known attacks on this protocol have been successfully found, the artificial static analysis is more complicated. Afterwards, many improved methods have been developed to verify more complex protocols^22–24^. In 2014, Henda proposed a general method to model intruder behavior^25^. That is, intruders can dynamically analyze intercepted messages and respond, which improves the automation of SPIN analysis of security protocols. However, there is model redundancy in the method, which can easily result in state explosion^26^. Since then, some scholars have improved the method^27^. Looking at the application of SPIN in the analysis and verification of security protocols, there is no specific research on the security of the protocol with SPIN in a composition protocol environment.

In summary, the contributions of the specific work are as follows.

(1) The formal description of the composition protocol operation semantics and composition protocol attack suitable for SPIN is given on the basis of the operation semantics of Ref.^28^ and Ref.^25^. And the weak consistency and the secrecy security properties of the composition protocol are formally defined.

(2) With the semantic model, a formal analysis method of composition protocol based on SPIN model checker is proposed.

(3) The intruder model is formally described, and the message specification method for field detection and component identification is presented to alleviate the state explosion problem, and the general intruder model in Ref.^25^ is optimized.

(4) The composition of NSB and NSL protocols is used as an example to carry out the detailed modeling and a known attack on the protocol update scenario is discovered successfully, which provides an idea for composition protocol security analysis with SPIN tools.

The rest of this paper is organized as follows: “Composition Protocol Analysis Method” describes the method of verifying compositional protocols based on SPIN analysis. “Semantic Model and Property Specification of Composition Protocol” gives the formal definition of the semantic model and property specification of the composition protocol. In “Promela Model”, the protocol used in this article is introduced, and the Promela compositional modeling process of the protocol is described. In “Experiment Results”, the experimental results are given. “Summary and Future Work” draws conclusions and looks forward to the next research work.

## Composition Protocol Analysis Method

**Figure 1:**
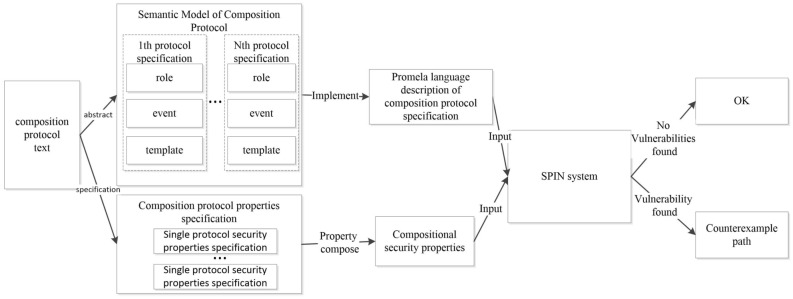
Analysis steps on composition protocol with the SPIN.

Aiming at the complexity of protocol security property verification in a composition protocol environment, we propose a method to analyze and verify the composition protocol using the SPIN model checker, referring to the method for verifying the composition protocol in Ref.^28^. The specific steps are as follows:

(1) The operational semantic model of the composition protocol system is established, and multiple roles are added to the model to intuitively describe the composed operation of multiple protocols.

(2) From the perspective of the protocol agent, the security attributes such as authentication and secrecy of the independent protocol are formalized into the agent’s declaration event, and the global security properties are expressed through the local security properties.

(3) The semantic model of the composition protocol is transformed into the SPIN system modeling language Promela, and the protocol role and the security properties from the protocol are applied as input. The independent security properties and the compositional security properties are verified through the SPIN tool to obtain possible counterexamples of composition protocol attacks. The steps to analyze the security of composition protocol with the SPIN are shown in Fig.1.

## Semantic Model and Property Specification of Composition Protocol

### Formal definition of protocol set

The protocol description usually includes variables, functions, structures that express messages, and a series of protocol events^25^. To formally define the security protocol, we assume that represents a set of type variables, and three possible variable types: agent (E), random number (N) and key (K) are mainly considered. The various types are formally defined based on the Backus paradigm as follows:

$$\begin{aligned} \begin{array}{l} \nu {:}{:} {=} \mathrm{{ E | N | K}}\\ \mathrm{{E }}{:}{:} {=} \mathrm{{ A}}|\mathrm{{B}}| \cdot \cdot \cdot |\mathrm{{I}}\\ \mathrm{{N }}{:}{:} {=} {\mathrm{{N}}_0}|{\mathrm{{N}}_1}| \cdot \cdot \cdot |{\mathrm{{N}}_n}\\ \mathrm{{K }}{:}{:} {=} \mathrm{{ PK}}|\mathrm{{SK}}|\mathrm{{SSK}} \end{array} \end{aligned}$$

Agent type E includes honest agents (AgentH) such as A, and an intruder agent represented by I; random number type N includes random numbers generated by agents such as $\mathrm {N}_{0}$ and $\mathrm {N}_{1}$; The key type K includes a public key PK, a private key SK, and a shared key SSK, which can be mapped into the agents through a fixed function. The message M can be a variable or a constant, which includes an atomic message m, a common message $\{\mathrm{M}_1, \mathrm{M}_2\}$ composed of multiple messages, and a message $\{\mathrm{M}_1\}\mathrm{K}$ encrypted or signed with a key. The specific definition is

$$\begin{aligned} \begin{array}{l} m{:}{:} {=} \mathrm{{ E}}|\mathrm{{N}}|\mathrm{{K}}\\ \mathrm{{M }}{:}{:} {=} \mathrm{{ m}}|\left\{ {\mathrm{{M}}_{1},{\mathrm{{M}}_2}} \right\} |\left\{ {{\mathrm{{M}}_1}} \right\} \mathrm{{K}} \end{array} \end{aligned}$$

The protocol set and its related definitions are given below:

#### Definition 1

**Role (r)**   $\mathrm{{r}} = (\nu ,\ell )$is a two-tuple consisting of the role’s variable set $\nu$ and the event index list $\ell$. The event index list maps the sequence of events that the role should execute.

#### Definition 2

**Event** ($\varepsilon$)   Event $\varepsilon$ is the action of the protocol role when the protocol is executed, which is defined as:

$$\begin{aligned} \varepsilon {:}{:} {=} \mathrm{{ send(r,M,r}}'\mathrm{{)|recv(r,M,r}}'\mathrm{{)|claim(r,c,r}}'/\mathrm{{t)|}}{\varepsilon _ \downarrow } \end{aligned}$$

Where $\mathrm{{send(r,M,r}}'\mathrm{{)}}$ represents role r sending message M to role $\mathrm{{r}}'$, $\mathrm{{recv(r,M,r}}'\mathrm{{)}}$represents role r receiving message M sent by role $\mathrm{{r}}'$. $\mathrm{{claim(r,c,r}}'/\mathrm{{t)}}$ means role r performs a security assertion event. Transparent event ${\varepsilon _ \downarrow }$ is an internal action event of the agent that is transparent to the intruder and does not affect the protocol interaction. It is defined as:

$$\begin{aligned} {\varepsilon _ \downarrow } = \mathrm{{start(r,M,r}}'\mathrm{{)|comt(r,M,r}}'\mathrm{{)}} \end{aligned}$$

Where $\mathrm{{start(r,M,r}}'\mathrm{{)}}$ indicates that role r initiates a conversation with role $\mathrm{{r}}'$, accompanied message M. $\mathrm{{comt(r,M,r}}'\mathrm{{)}}$ indicates that role r submits a session with role $\mathrm{{r}}'$, accompanied message M.

#### Definition 3

**Message template** ($\varpi$)   The message template $\varpi = \mathrm{{t}}{\mathrm{{p}}_{1}}\mathrm{{,t}}{\mathrm{{p}}_{2}},...\mathrm{{t}}{\mathrm{{p}}_\mathrm{{n}}}$ is a sequence of type variables conforming to the message M specified by the specific protocol, and tpi is the message type. It can be obtained by the field check function type(M).

#### Definition 4

**Interaction relationship** ($\prec$)   The interaction relationship $\prec : \varepsilon \times \varepsilon$ is defined as

$$\begin{aligned} \begin{array}{l} \forall {\varepsilon _1},{\varepsilon _2} \in \varepsilon :{\varepsilon _1} \prec {\varepsilon _2} \Leftrightarrow \\ \exists \mathrm{{r,r}}',\mathrm{{M}}:{\varepsilon _1} = \mathrm{{send(r,M,r}}') \wedge {\varepsilon _2} = \mathrm{{recv(r}}'\mathrm{{,M,r)}} \end{array} \end{aligned}$$

#### Definition 5

**Associated role** ($\sim$)^28^   Let ${{\mathscr {Q}}_\mathrm{{i}}}$ be a protocol, and the symbol $\preccurlyeq$ represent a symmetric, reflexive, and transitive interaction closure $\prec$. Roles r and $\mathrm{{r}}'$ are related roles $\mathrm{{r}} \sim \mathrm{{r}}'$, if and only if

$$\begin{aligned} \exists {\varepsilon _1}{\text {,}}{\varepsilon _2}:{\varepsilon _1} \in {\varepsilon ^ * }({\text {r}}) \wedge {\varepsilon _2} \in {\varepsilon ^ * }({\text {r}}') \wedge {\varepsilon _1} \preccurlyeq {\varepsilon _2}, \end{aligned}$$

where ${\varepsilon ^ * }(\mathrm{{r}})$ represents the sequence of events of role r.

#### Definition 6

**Independent protocol** (${\mathscr {Q}}$)   According to the above definition, the independent protocol can be defined as a triplet, namely ${\mathscr {Q}} = ({\mathrm{{R}}^ * },{\varepsilon ^*},{\varpi ^ * })$, if and only if:

$$\begin{aligned} \begin{array}{l} \forall \mathrm{{m, n}} \in :\mathbb {N}\\ {\varepsilon ^ * } = \{ {\varepsilon _0},{\varepsilon _1},...{\varepsilon _n}\} \wedge {\varpi ^ * } = \{ {\varpi _0},{\varpi _1}...{\varpi _n}\} \wedge \\ {\mathrm{{R}}^ * } = \{ {\mathrm{{r}}_0},{\mathrm{{r}}_1}...{\mathrm{{r}}_m}\} \wedge \\ \forall {\mathrm{{r}}_\mathrm{{i}}}\mathrm{{,0}} \le \mathrm{{i}} \le \mathrm{{m: }}\exists {\mathrm{{r}}_\mathrm{{j}}},0 \le \mathrm{{j}} \le \mathrm{{m}}:{\mathrm{{r}}_\mathrm{{i}}} \sim {\mathrm{{r}}_\mathrm{{j}}} \end{array} \end{aligned}$$

$\mathbb {N}$ is an arbitrary positive integer greater than 1. In other words, for every role in a protocol there is always another role within that protocol that becomes a related role. That is, all roles in the protocol conform to the interaction relationship.

#### Definition 7

**Protocol set** ($\Pi$)   $\Pi = \{{\mathscr {Q}}_{1},{\mathscr {Q}}_\mathrm{{2}},...{\mathscr {Q}}_\mathrm{{n}}\}$ is a protocol set, where $n \in \mathbb {N}$, for $\forall 1 \le i \le n, {{\mathscr {Q}}_\mathrm{{i}}} \in \Pi$ is an independent protocol in the protocol set $\Pi$.

To define a composition protocol attack, we denote the attack that violates the security property c in a single protocol ${\mathscr {Q}}$ as $\mathrm{{attack(}}{\mathscr {Q}}\mathrm{{,c)}}$, and the attack that violates the security property c in the protocol set $\Pi$ as $\mathrm{{attack(}}\Pi \mathrm{{,c)}}$. Therefore, based on the definition in Ref. ^12^, the definition of the composition protocol attack is expanded as follows.

#### Definition 8

**Composition protocol attack** ($\Pi \mathrm{{ - attack}}$)   There is a composition protocol attack $\Pi \mathrm{{ - attack}}\left( {\Pi \mathrm{{,c}}} \right)$ that violates the security property c in the protocol set $\Pi$ including protocol ${{\mathscr {Q}}_\mathrm{{i}}}$, if and only if

$$\begin{aligned} \begin{array}{l} \mathrm{{attack(}}\Pi \mathrm{{,c)}} \wedge \\ \forall \Pi \mathrm{{'}} \subset \Pi :\lnot \mathrm{{attack(}}\Pi \mathrm{{',c)}} \wedge \\ \exists {{\mathscr {Q}}_\mathrm{{i}}} \in \Pi :\lnot \mathrm{{attack(}}{{\mathscr {Q}}_\mathrm{{i}}}\mathrm{{,c)}} \end{array} \end{aligned}$$

In other words, when an independent protocol in a protocol set does not violate a certain property, but such violation occurs in the execution environment of the entire protocol set, and each protocol in the protocol set participates in this violation, then an attack that results in a violation of this property is a composition protocol attack.

### Formal definition of intruder

The Dolev-Yao model^29^ is an intruder model widely adopted in the formal analysis and verification of security protocols. The main contents are as follows:

(1) The intruder can eavesdrop and intercept all messages on the network.

(2) The intruder knows the identity and the public key of the agents participating in the protocol.

(3) The intruder can participate in the operation of the protocol as a legal agent or pretend to be other participants in the protocol.

(4) The intruder can store the intercepted messages and can also expand his knowledge set with the intercepted messages.

(5) The intruder can replay the message or apply his own knowledge to forge the message.

The intruder in this paper is also based on the DY model. The relevant symbols for the formal description of the intruder are defined as follows:

**Table:** 

symbol	meaning
KN	Intruder’s knowledge set
Net	The network monitored by the intruder
Chan	Communication channel
Invade	Intruder behavior
BS	Intruder behavior pattern
$\square$	Always
$\rightarrow$	Sequential operator
$\vee$	Logical or
$\vdash$	Deduced

$$\begin{aligned} \begin{aligned}{}&{\text {KN }}{:}{:} {=} {\text { KN}}\left( {\text {m}} \right) \cup {\text {KN}}\left( {\text {C}} \right) \\&{\text {Net }}{:}{:} {=} {\text { Cha}}{{\text {n}}_1} \cup {\text {Cha}}{{\text {n}}_2} \cup \cdot \cdot \cdot \cup {\text {Cha}}{{\text {n}}_{\text {n}}} \\&{\text {Chan }}{:}{:} {=} {{\text {M}}_1} \cup {{\text {M}}_2} \cup \cdot \cdot \cdot \cup {{\text {M}}_{\text {n}}} \\& {\text {Invade }}{:}{:} {=} {\text { Intercept}} \cup {\text {Analysis}} \cup {\text {Forge}} \cup {\text {Send}}; \\& {\text {BS }}{:}{:} {=} \square {\text {[(Intercept}} \rightarrow {\text {Analysis}}) \vee ({\text {Forge}} \rightarrow {\text {Send}}){\text {]}}\\ \end{aligned} \end{aligned}$$

KN is composed of the atomic knowledge set KN(m) and the component knowledge set KN(C). The component knowledge set is used to store the encrypted components that the intruder cannot learn. For a simple protocol that does not contain nested encryption or multiple encryption components in the protocol message, it can be expanded into a message library. Net can be abstracted into each channel Chan, where Chan is defined as the set of messages currently stored in the channel. Invade represents the intruder’s behavior which consists of intercepting messages (Intercept), analyzing messages (Analysis), forging messages (Forge), and sending messages (Send). BS describes the execution sequence of the intruder’s actions. The specific algorithm description of the intruder’s behavior is given below.

**Figure 2:**
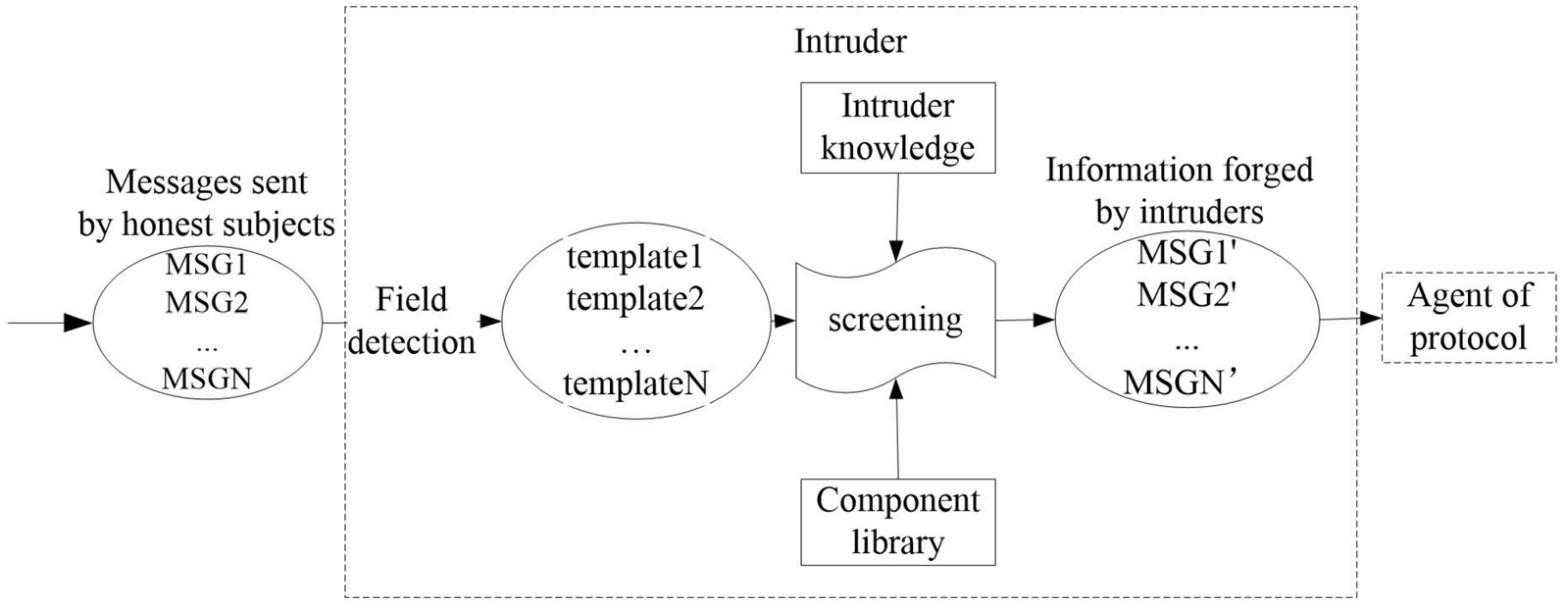
Field detection and component identification application diagram.

- Intercept behavior
  According to the DY model, the intruder can intercept all messages in the monitoring network. The specific implementation is shown in Algorithm 1. 
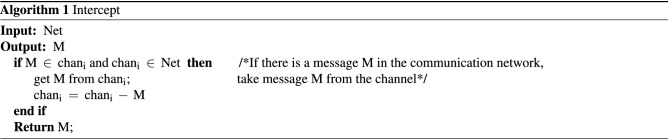
- Analysis behavior
  When the intruder receives or intercepts a message, it will deconstruct the message and expand the unknown message to the knowledge set KN with the analysis behavior. The specific implementation is shown in Algorithm 2. 
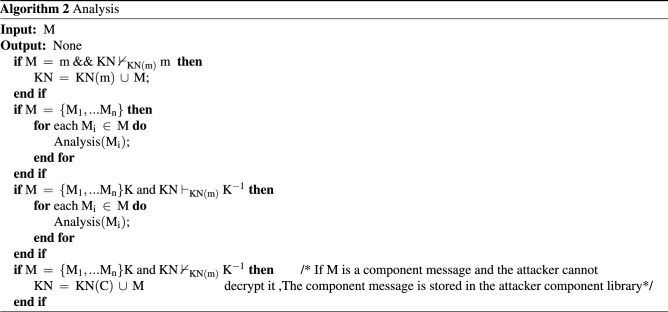
- Forge behavior
  The current non-static method of message specification through type detection is to utilize the type label attached to the message to determine the type of the message in the channel, thereby reducing the value range of various types of variables. This method can be roughly divided into two categories. One uses the intercepted message as a message template $\varpi$ to forge the same type of message^30^; the other considers the current template requirements of each protocol entity to forge a message that meets the needs of the entity type. Since the number of entities in a composition protocol environment is far more than that in a single-protocol environment, we choose the intercepted message type as the message template.
  When the intruder adopts the Forge behavior, the intercepted message is converted into a message template $\varpi$ through the field detection method, and a new message conforming to $\varpi$ is forged according to his own knowledge set KN. If the content of the component library is not zero, the intruder can also combine the atomic knowledge set and the component knowledge set to priori judgment which messages can be forged and conform to the current message template $\varpi$.If all the component types are matched successfully, the intruder can use the encrypted component in the component library and atomic knowledge set to forge valid messages. Compared with the method in Ref.^25^ that first forges the message and then judges whether the message can be constructed, the method in this paper can effectively avoid the generation of redundant messages and further reduce the number of states in protocol verification. The above two points are the message specification method based on field detection and component identification proposed in this paper to effectively alleviate the problem of state explosion in a composition protocol environment. The application during the execution of the protocol is shown in Fig.2
  The specific implementation is shown in Algorithm 3. 
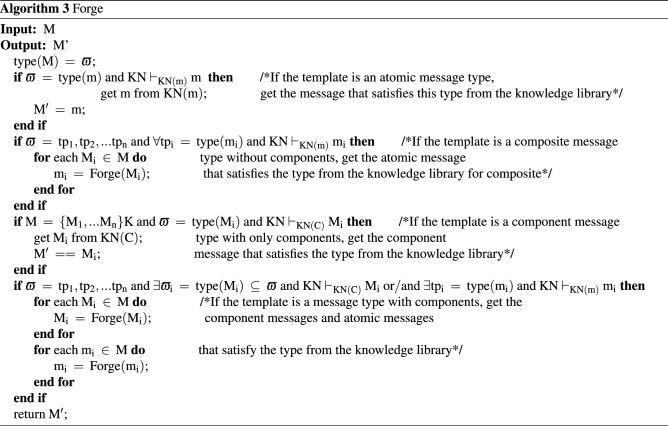
- Send behavior
  When the intruder employs the send behavior, a message can be sent to the monitoring network. The specific implementation is shown in Algorithm 4. 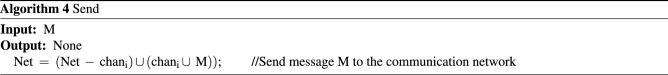

According to the above definition of symbols, the intruder model in this paper can be formally defined as a quadruple (KN, Invade, BS, Net). That is, the intruder model is composed of his knowledge set, behavior ability, behavior pattern, and monitoring network.

###  Operational semantics

Since Ref.^28^ and Ref.^25^ have made many excellent good contributions on the operational semantics of security protocols, we refer to their contents to describe and extend security protocol operational semantics suitable for guiding SPIN modeling and security properties verification in the context of composition protocol, as follows:

#### Definition 9

**Rounds (run)**   $\mathrm{{run}} = (\theta ,\mathrm{{r,}}{{{\sigma }}^\mathrm{{*}}}\mathrm{{\{ r\} )}}$ is a triplet consisting of round identification $\theta$ , protocol role r, and permutation set ${{{\sigma }}^\mathrm{{*}}}\mathrm{{\{ r\} }}$, where $\mathrm{{\sigma }}$ represents a permutation ${{\sigma :}}\nu \mathrm{{(r)}}{ \mapsto _\varpi }\mathrm{{E}} \cup \mathrm{{N}} \cup \mathrm{{K}}$, and ${{{\sigma }}^\mathrm{{*}}}\mathrm{{\{ r\} }}$ represents the set of all possible permutations of the role r. A round can uniquely identify a process performed by an entity according to a certain protocol rule^31^.

#### Definition 10

**Role instantiation (Inst)**   Protocol ${{\mathscr {Q}}_\mathrm{{i}}}$ is selected in protocol set $\Pi$, $\mathrm{{r}} \in {{\mathscr {Q}}_\mathrm{{i}}}$ . Then an instance of role r is represented by a quadruple $(\theta ,\mathrm{{r,j,}}\sigma \{ \mathrm{{r}}\} )$. j is the index number of the role event index list (starting from 1), which means that the instance will execute the j-th event $\mathrm{{r[j]}}$ in next step. $\sigma \{ \mathrm{{r}}\}$ represents the permutation set of the current role r.

#### Definition 11

**Labelled transition system (LTS)**   The labelled transition system LTS is a quaternion $(\mathrm{{S}},\varepsilon , \rightarrow ,{\mathrm{{s}}_0})$, where S is a state set, $\varepsilon$ is the transition event between states, $\rightarrow$ is the transition relationship between states, and ${S_0}$ represents the initial system state. Let F denote the currently active instance set, KN denote the current intruder’s knowledge set. ${S_0}$ the current system state can be represented by $\mathrm{{s = < KN,F > }}$. Assuming that the initial value of the intruder’s knowledge set is $\mathrm{{K}}{\mathrm{{N}}_\mathrm{{0}}}$, then the initial state of the entire system is ${\mathrm{{s}}_0} = \left\langle {\mathrm{{K}}{\mathrm{{N}}_\mathrm{{0}}},\emptyset } \right\rangle$.

#### Definition 12

**Round identifier set (RIDs)**   $\mathrm{{RIDs(F)}}$ is the round identifier set, which is adopted to record all round identifiers in the current state, namely $\mathrm{{RIDs(F)}} = \{ \theta |(\theta ,\mathrm{{r,j,}}\sigma \{ \mathrm{{r}}\} )\in \mathrm{{F}}\}$.

#### Definition 13

**Match**   The matching predicate Match can help in judging whether the message structure of the incoming message matches the message expected by the agent. The specific definition is as follows:

$$\begin{aligned}\begin{array}{l} \mathrm{{Match(inst,x,M,inst}}') \Leftrightarrow \mathrm{{inst}} = (\theta ,\mathrm{{r,j,}}\sigma \{ \mathrm{{r}}\} ) \wedge \mathrm{{inst}}' = (\theta ,\mathrm{{r,j,}}\sigma '\{ \mathrm{{r}}\} ) \wedge \\ {{\sigma }}'\{ \mathrm{{r}}\} = \sigma \{ \mathrm {r}\} \cup \sigma \mathrm {(x)} \wedge \mathrm{{type(x) = type(M) = }}\varpi \wedge \\ \mathrm{{\sigma (x) = M}} \end{array} \end{aligned}$$

where $\sigma {\mathrm{(x)}}$ is applied to instantiate message x.

#### Definition 14

**Well-formed** ($\Psi$)   For a role r and a state $\mathrm{{s}} \in \mathrm{{S}}$, it is said that the variable instantiated by ${{\sigma }}$ is well-formed $\Psi {{(\sigma )}}$ for the state s, if and only if,

$$\begin{aligned}\begin{array}{l} \sigma (\mathrm{{N}}):\mathrm{{ V(N)}}{ \mapsto ^\sigma }_\varpi \mathrm{{N'}} \wedge \\ \mathrm{{ N'}} \notin {{{\sigma }}^\mathrm{{*}}}(\mathrm{{N}}) \end{array} \end{aligned}$$

where ${{{\sigma }}^\mathrm{{*}}}(\mathrm{{N}})$ represents the set of all instantiated random numbers. In other words, the permutation function $\sigma$ will not instantiate multiple random variables of r to the same random value.

To track the changes of the role instance during the execution of the protocol set. For a role instance $(\theta ,\mathrm{{r,j,}}\{ \mathrm{{r}}\} )$, a mapping function $\mathrm{{Th}}(\theta )$ is defined to map the round identifier to a role instance^25^. The specific definition is as follows.

$$\begin{aligned} \forall n \in \mathbb {N}:\mathrm{{Th}}[{{\theta }} \mapsto (\theta ,\mathrm{{r,j}},\sigma \{ \mathrm{{r}}\} )]\mathrm{{(n)}}{:} {=} \left\{ \begin{array}{l} (\theta ,\mathrm{{r,j,}}\sigma \{ \mathrm{{r}}\} ) \quad \mathrm{{if}} \;\mathrm{{n}} = \theta ;\\ \mathrm{{Th(n)}}\qquad \qquad \mathrm{{else}}. \end{array} \right. \end{aligned}$$

**Figure 3:**
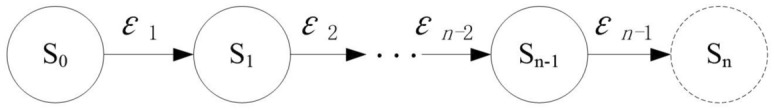
System state transition.

When the protocol specification behavior is executed, the intruder’s knowledge set is continuously updated, and the instance in F is updated with the $\mathrm{{Th}}(\theta )$ function, so that the system state s continuously transit to the next state under the influence of the transition event by relying on the transition relationship $(\xrightarrow {\varepsilon })$. The entire composition protocol interaction process is formally described. as shown in Fig.3.

**Figure 4:**
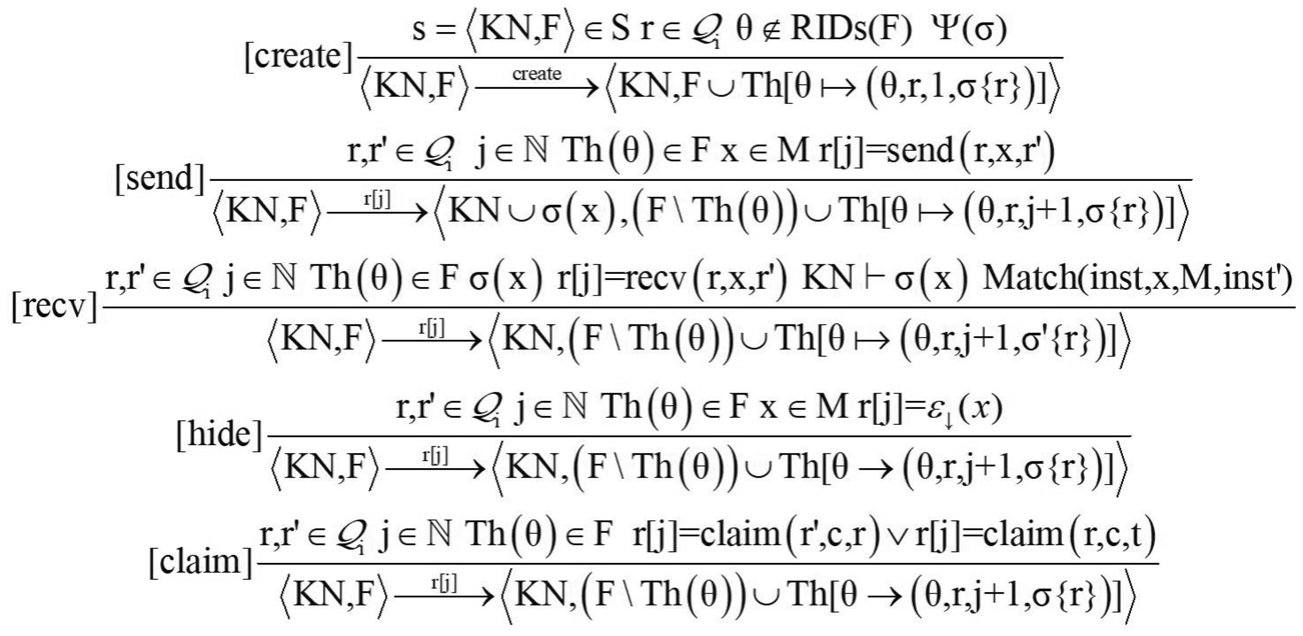
Transition relationship.

Three behavioral rules of creation (create), sending (send), and receiving (recv) of the protocol entity are given below to formally describe the protocol operation process in a composition protocol environment (see Fig.4), among which the hide and claim rules are used to describe security properties specification.

### Security properties

As mentioned above, we use the labelled transition system $(\mathrm{{S}},\varepsilon , \rightarrow ,{s_0})$ to describe the process of composition protocol execution and transform it into the system state transition process ${s_0}\xrightarrow {{{\varepsilon _0}}}{s_1}\xrightarrow {{{\varepsilon _1}}}{s_2}...\xrightarrow {{{\varepsilon _{n - 1}}}}{s_n}$. Therefore, a transition event sequence ${\varepsilon _0}{\varepsilon _1}...{\varepsilon _{n - 1}}{\varepsilon _n}$ can be applied to represent the event execution process of a composition protocol, denoted as trace $\tau$. The set of all traces in the protocol set is denoted as $\Gamma (\Pi )$. The secrecy and the authentication of the protocol are mainly considered. To effectively define the compositional security properties in the composition protocol system, we integrate the security properties into the protocol specifications of each protocol from the perspective of the protocol agent, and formally defines it as agent security asserting events. Through the partial security properties to determine whether the security properties of the expected protocol and even the entire composition protocol are satisfied, which is feasible for the parallel combination method. The assertion event can be implemented by the claim rule in Fig.4.

#### Definition 15

**Honest**   Let protocol ${{\mathscr {Q}}_\mathrm{{i}}} \in \Pi$ have roles r and $\mathrm{{r'}}$, $\mathrm{{r'}} \sim \mathrm{{r}}$. Instantiation of auxiliary predicate honesty definition are:

$$\begin{aligned} \mathrm{{honest}}(\theta ,\mathrm{{r,j,}}\sigma \{ \mathrm{{r}}\} )\Leftrightarrow \sigma (\mathrm{{r}}),\sigma (\mathrm{{r}}') \subseteq \mathrm{{Agen}}{\mathrm{{t}}_\mathrm{{H}}} \end{aligned}$$

Honest means that the correspondents and entities expected in the role instance honestly execute the protocol according to the protocol specification.

The secrecy of the secret item t is defined as follows:

#### Definition 16

**Secrecy**   Let the protocol set $\Pi$ have the role r, and t is the secret item of the role r. The secret assertion event $\gamma = \mathrm{{claim(r,secret,t)}}$ is established if and only if:

This definition means that the assertion of secrecy is established if and only if it is satisfied for all traces in the protocol set $\Pi$ : the role in each round can be matched as an honest agent, when it is declared that a certain message item t in the protocol set $\Pi$ is secret, the intruder cannot infer the content of the message from his own knowledge set before the end of the protocol.

The authentication is defined as follows:

#### Definition 17

**Authentication**   Let protocol ${{\mathscr {Q}}_\mathrm{{i}}} \in \Pi$ have roles r and $\mathrm{{r'}}$, $\mathrm{{r'}} \sim \mathrm{{r}}$. For any two events $\mathrm{{comt(r,x,r')}}$ and $\mathrm{{start(r',y,r)}}$ that are transited by the hide rule, the authentication assertion event $\gamma = \mathrm{{claim(r',wagree,r)}}$ holds if and only if:

$$\begin{aligned} & \forall \tau \in \Gamma (\Pi ),\forall {\text{i}},1 \le {\text{i}} \le |\tau ^{*} |: \\ & \tau ^{*} [{\text{i}}] = {\text{comt}}({\text{r}},{\text{x}},{{r^{\prime}}}) \wedge {\text{loc}}({\text{comt}}({\text{r}},{\text{x}},{{r^{\prime}}})) = (\theta ,{\text{r}},\sigma ^{*} \{ {\text{r}}\} ) \wedge {\text{loc}}(\gamma ) = (\theta ,{\text{r}},\sigma ^{{\text{*}}} \{ {\text{r}}\} ) \Rightarrow \\ & \exists {\text{j}}1 \le {\text{j}} \le {\text{i}}:\tau ^{*} [{\text{j}}] = {\text{start}}({{r^{\prime}}},{\text{y}},{\text{r}}) \wedge {\text{loc}}({\text{start}}({{r^{\prime}}},{\text{y}},{\text{r}})) = (\theta ^{\prime},{{r^{\prime}}},\sigma ^{*} \{ {{r^{\prime}}}\} ) \\ \end{aligned}$$

Authentication refers to a kind of identity confirmation of the parties in the protocol to ensure that the communication party is the expected legal agent. At present, there are many classifications of authenticity. We extend the weak consistency (wagree)^32^ pointed out by Lowe into a composition protocol environment. That is, in protocol ${{\mathscr {Q}}_\mathrm{{i}}} \in \Pi$, every time the responder completes a conversation with the initiator, the initiator must have initiated a conversation with the responder before this, and vice versa. To formally express this property, we refer to the method in Ref.^25^, with two special event actions start and comt extend role events that are transparent to the intruder. For a trace $\tau$ of the protocol set $\Pi$, ${\tau ^ * }$ is used to represent the sequence of events to be transited by the hide rule, $|{\tau ^ * }|$ represents the maximal length of the ${\tau ^ * }$ sequence, and ${\tau ^ * }[\mathrm{{i}}]$ represents the i-th element of ${\tau ^ * }$. $\mathrm{{loc(c)}}$ locates the execution round run of the role event c.

## Promela Model

### Protocol case

Security protocols are often found to be broken in some way after deployment. This problem can be resolved through protocol updates. The updated protocol is actually the second protocol, which is very similar to the first protocol and shares the same key structure. This situation makes it possible for composition protocol attacks to occur. We apply protocol case NSB (Needham-Schroeder: Broken) protocol^12^ and NSL(Needham-Schroeder-Lowe) protocol to verify composition protocol attacks.

**Figure 5:**
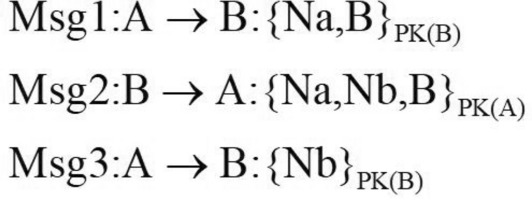
NSB protocol.

The NSB protocol is a flawed authentication protocol that aims to achieve mutual authentication between two agents. The protocol is implemented by a public key encryption system at the cryptographic level. Like the attack described in Ref.^21^, this protocol is vulnerable to the man-in-the-middle attacks. The flow of the NSB protocol is shown in Fig.5.

**Figure 6:**
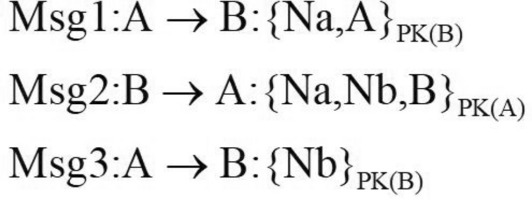
NSL protocol.

The NSL protocol is an improvement of the classic NSPK protocol by David G. Lowe, which has been proven to be secure when it runs on its own. The flow of the NSL protocol is shown in Fig.6.

### Model assumptions

Based on the Dolev-Yao model, we first give the assumptions that need to be met during the modeling process:

(1) Assume that the cryptographic algorithms used in the protocol are perfect.

(2) The format of the message in the protocol is standardized, that is, the message will be received only if it meets the message format required by the receiver.

(3) There are multiple protocol instances running in an untrusted shared network at the same time.

(4) For public key cryptosystems, the intruder will not use his own public key when forging a message.

The Promela modeling process of the composition protocol is divided into three stages, namely that protocol agent modeling, intruder modeling, and security property characterization. The modeling phase of the protocol agent is relatively simple, and only needs to describe the interaction process between honest agents participating in the protocol, which can be automatically generated by a higher-level specification language. The latter two stages are common, and the generated model can be used for different protocols after modification.

### Protocol agent model

The first step in the modeling of the protocol agent is to model the communication channels between the agents. The channel is an abstraction of the communication network between agents during modeling. To simplify the model, we only define a synchronization channel network, so the length of the channel should be equal to the longest message length in all the protocols that participate in the execution. An Msgi field is added to the front of the message to identify the message type in the channel. Note $\mathrm{{Len(M)}}$ is the length of the message M, $\mathrm{{Len(NSB|NSL)}}$ is the maximum message length in the NSL protocol and the NSB protocol, m is the atomic message, and N is the number of message types in the protocol. It is defined as follows:

$$\begin{aligned}&\mathrm{{Len(M) = }}\left\{ \begin{array}{l} 1,\qquad \qquad \qquad \qquad \mathrm{{M = = m;}}\\ \mathrm{{Len(}}{\mathrm{{M}}_\mathrm{{1}}}\mathrm{{) + Len(}}{\mathrm{{M}}_\mathrm{{2}}}\mathrm{{), \;M = = \{ }}{\mathrm{{M}}_\mathrm{{1}}}\mathrm{{,}}{\mathrm{{M}}_\mathrm{{2}}}\} ;\\ \mathrm{{Len(}}{\mathrm{{M}}_\mathrm{{1}}}\mathrm{{) + Len(}}{\mathrm{{M}}_\mathrm{{2}}}\mathrm{{),\; M = = }}\left\{ {{\mathrm{{M}}_\mathrm{{1}}}} \right\} {\mathrm{{M}}_\mathrm{{2}}} \end{array} \right. \\&\quad \mathrm{{Len}}(\mathrm{{NSB}}|\mathrm{{NSL}}) = Max\left\{ {\bigcup \nolimits _{\mathrm{{i = 1}}}^\mathrm{{N}} {\mathrm{{Len}}(\mathrm{{Msgi}})} } \right\} \end{aligned}$$

The channel capacity is $\mathrm{{Len(NSB|NSL) }} + 1 = 6$, as the protocol must add its own identity to the message. Then the channel Promela modeled by the NSB and the NSL composition protocol is defined as follows:

$$\begin{aligned} \mathrm{{chan network = }}\left[ \mathrm{{0}} \right] \mathrm{{ of }}\left\{ {\mathrm{{mtype, mtype, mtype, mtype, mtype, mtype}}} \right\} ; \end{aligned}$$

The second step in the modeling of the protocol agent is to define the limited name set in the protocol, including the different identifiers, entities, keys, and random numbers in the protocol. The Promela name set used in this paper is defined as follows:

$$\begin{aligned} \mathrm{{mtype = }}\left\{ {\mathrm{{NULL, Msg1, Msg2, Msg3, A, B, I, N3, N2, N1, N0, Ni, PKa, PKb, PKi}}} \right\} ; \end{aligned}$$

Here NULL represents a placeholder, which can be used to fill empty fields. Since the entire mtype set is decremented from NULL to 1, the NULL value is equal to the size of the name set. $\mathrm{{N0,}}...\mathrm{{,N3}}$ represent the random numbers generated by honest agents A and B in the protocol, and Ni represents the random number generated by the intruder. $\mathrm{{Msg1,Msg2,Msg3}}$ represent the message types in the composition protocol.

**Figure 7:**
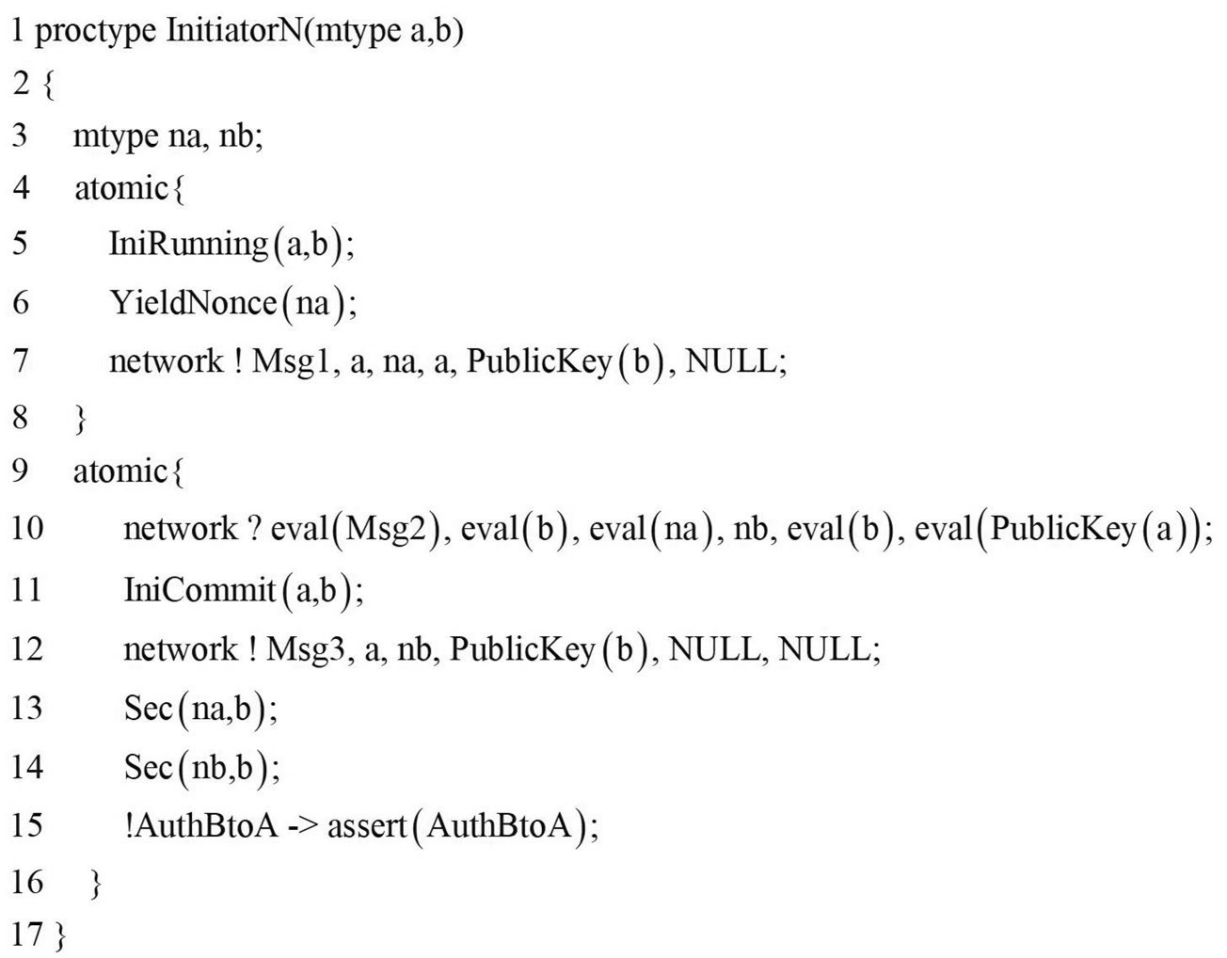
The initiator process of the NSL protocol.

The next step in the modeling of the protocol agent is the modeling of the protocol role. The realization of the protocol role is represented by the Promela process and is instantiated through certain parameters. As this paper verifies the security of the composition protocol, the creation of two initiator processes represents the initiator role of the NSL protocol and the NSB protocol, and the two responder processes represent the responder role of the NSL protocol and the NSB protocol. As shown in Fig.7, the $\mathrm{{InitiatorN}}$ process is used to represent the initiator role of the NSL protocol, and the process parameters a and b are respectively used to instantiate the initiator role of the protocol and its communicating party, where $\mathrm{{IniRunning(a,b)}}$ and $\mathrm{{IniCommit(a,b)}}$ are special events describing the properties of authentication, $\mathrm{{Sec(na,b)}}$ and $\mathrm{{Sec(nb,b)}}$ are local secrecy assertions, which will be described in detail in the security properties implementation section later.

Lines 7, 10, and 12 of Fig.7 show the three communication events performed by the initiator role of the NSL protocol during the operation of the protocol, that is, two events are sent, and one event is received. And the entity’s receive statement and the next send statement are placed an atomic statement means that these two operations are completed in one atomic step, which can effectively reduce the number of states. The first field Msg in the message structure is used to identify the type of the message in the channel, and the second field indicates the sender of the message. The Macro PublicKey is a mapping function from entity to public key, as shown below.

$$\begin{aligned} \# \mathrm{{define PublicKey}}\left( \mathrm{{x}} \right) \mathrm{{ x - }}\left( {\mathrm{{A - PKa}}} \right) \end{aligned}$$

The eval() function in the message receiving statement is used to force the message field to match the current value of the local or global variable in the receiving statement. The inline function $\mathrm{{YieldNonce(d)}}$ is a random number generation function. When modeling a composition protocol, the same agent will participate in multiple rounds, thereby generating multiple random numbers and each random number must be unique. In this paper, the random number is not bound to the entity, and the random number is generated through an inline function to ensure that the instantiation of the random number is well-structured( ). This method is simple, and the model has a high degree of automation. $\mathrm{{YieldNonce(d)}}$ is implemented as follows:

$$\begin{aligned} & {\text{mtypenonce}} = {\text{N0}}; \\ & {\text{inlineYieldNonce}}\left( {\text{d}} \right)\{ \\ & \,\,\,{\text{d}} = {\text{nonce}}; \\ & \,\,\,{\text{nonce}}\,{\text{ + + }}; \\ & \} \\ \end{aligned}$$

**Figure 8:**
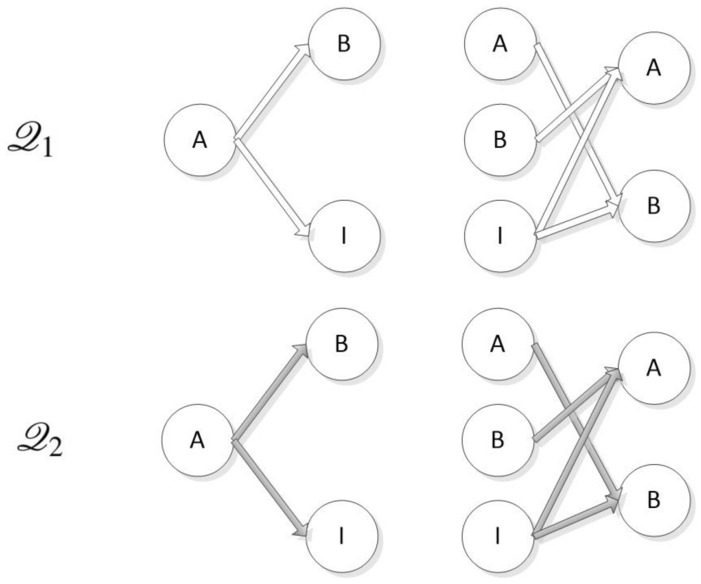
Agent interaction diagram.

The implementation of the responder process of the NSL protocol and the agents process of the NSB protocol is similar to the $\mathrm{{InitiatorN}}$ process. The format and type checking of the message are postponed to the process, which will not be repeated here. The last step in the modeling of the protocol body is to initialize all processes, which requires an init process. In this process, each initiator instance and each responder instance are represented by the introduction of process instance statements. In that the intruder may be an honest agent that has been compromised, it is necessary to consider the initiator initiating a conversation with the intruder in the init process. The agent A as the initiator participates in the protocol and the agent B as the initiator participates in the protocol is completely symmetrical. Therefore, we only instantiate the case where agent A is the initiator. Considering the possibility of parallel session attacks, both agents A and B need to be instantiated as responder processes. In addition, we are to model the combination of two protocols, and each entity also needs to choose the protocol. The main interaction is shown in Fig.8.

So, the initialization process of this paper is defined as follows:

$$\begin{aligned} \begin{array}{l} \mathrm{{init}}\\ \{\\ \;\;\;\;\mathrm{{atomic}}\{ \\ \;\;\;\;\mathrm{{if}} \qquad \qquad \qquad \qquad \qquad \;\mathrm{{if}}\\ \;\;\;\;{:}{:}\mathrm{{run}}\; \mathrm{{InitiatorO}}\left( {\mathrm{{A,B}}} \right) ; \qquad \mathrm{{{:}{:}run}}\; \mathrm{{ResponderO}}\left( \mathrm{{B}} \right) ;\\ \;\;\;\;\mathrm{{{:}{:}run}}\; \mathrm{{InitiatorO}}\left( {\mathrm{{A,I}}} \right) ; \qquad \;\;{:}{:}\mathrm{{run}}\; \mathrm{{ResponderO}}\left( \mathrm{{A}} \right) \mathrm{{;}}\\ \;\;\;\;\mathrm{{fi}}; \qquad \qquad \qquad \qquad \qquad \mathrm{{fi;}}\\ \;\;\;\;\mathrm{{if}} \qquad \qquad \qquad \qquad \qquad \;\mathrm{{if}}\\ \;\;\;\;{:}{:}\mathrm{{run}}\; \mathrm{{InitiatorN}}\left( {\mathrm{{A,B}}} \right) ; \qquad {:}{:}\mathrm{{run}}\; \mathrm{{ResponderN}}\left( \mathrm{{B}} \right) \mathrm{{;}}\\ \;\;\;\;{:}{:}\mathrm{{run}}\; \mathrm{{InitiatorN}}\left( {\mathrm{{A,I}}} \right) ; \qquad \; {:}{:}\mathrm{{run}}\; \mathrm{{ResponderN}}\left( \mathrm{{A}} \right) \mathrm{{;}}\\ \;\;\;\;\mathrm{{fi}}; \qquad \qquad \qquad \qquad \qquad \mathrm{{fi;}}\\ \qquad \qquad \qquad \qquad \qquad \qquad \mathrm{{run}}\;\mathrm{{Intruder();}}\\ {{\qquad \qquad \qquad \qquad \qquad \qquad \} }}\\ {{\;\;\qquad \qquad \qquad \qquad \qquad \} }} \end{array} \end{aligned}$$

As shown above, the initialization process must contain a process instance (Intruder) representing the intruder. The next section will explain the process definition of the intruder.

### Intruder model

The most important part of the formal analysis of the security protocol is the intruder modeling part. According to the previous formal definition of the intruder model, a powerful and efficient intruder model can be established. First, we need to define the intruder’s knowledge set, as shown below.

$$\begin{aligned} \begin{array}{*{20}{l}} {\mathrm{{bool Knows}}\left[ {\mathrm{{NULL}}} \right] \mathrm{{;}}}\\ {\mathrm{{bool ReverseKeys}}\left[ {\mathrm{{NULL}}} \right] \mathrm{{;}}}\\ {\mathrm{{typedef CON\{ }}}\\ \;\;\;\;{\mathrm{{mtype d1;}}}\\ \;\;\;\;{\mathrm{{mtype d2;}}}\\ \;\;\;\;{\mathrm{{mtype d3;}}}\\ \;\;\;\;{\mathrm{{mtype key;}}}\\ \}\\ {\mathrm{{CON CA}}\left[ 1 \right] \mathrm{{;}}} \end{array} \end{aligned}$$

We divide the intruder’s knowledge set into atomic knowledge set and component knowledge set. The atomic knowledge set is represented by Boolean arrays Know[] and Reversekeys[]. The array Know[] represents the atomic messages learned by the intruder, and the array Reversekeys[] means that the intruder has learned the decryption key of the corresponding key. The component knowledge set is represented by the array CA[], which is used to store encrypted components that the intruder cannot learn. The type of CA[] is a custom message structure CON.

The purpose of the component knowledge set proposed in this paper is to improve the versatility and efficiency of the model. The method in Ref.^25^ is mostly used for the formal verification of simple protocols. It is often difficult for complex protocols containing multiple encrypted blocks or nested encrypted messages. Relying on the entire message stored in the message library to forge this kind of message is more complicated. Using the component knowledge library instead of the message library can simplify this process and improve the versatility of the model. Since the message intercepted by the intruder is essentially a kind of knowledge containing redundant elements, for simple protocols, the component library can also be expanded into a message library to increase the degree of automation of the model. To reduce the number of states of the model in this paper, the size of the component library is set to 1, which is correct for the simple protocol, because the simple protocol does not need to obtain unknown component messages from two or more old messages.

**Figure 9:**
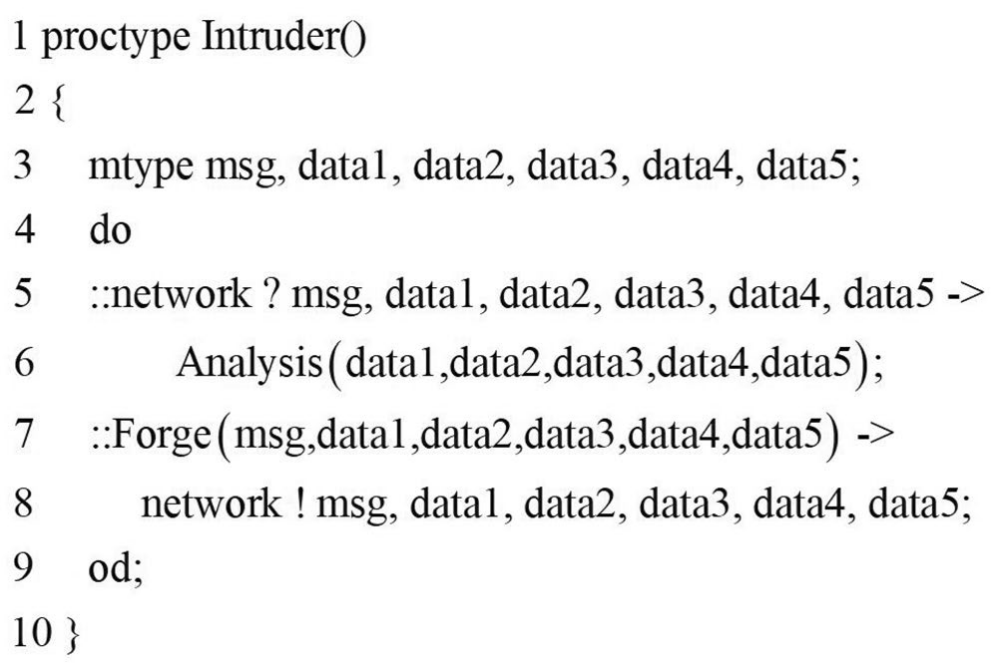
Intruder process.

According to the formal definition of the intruder in Section 3.2, the intruder process in this paper is established, as shown in Fig.9.

**Figure 10:**
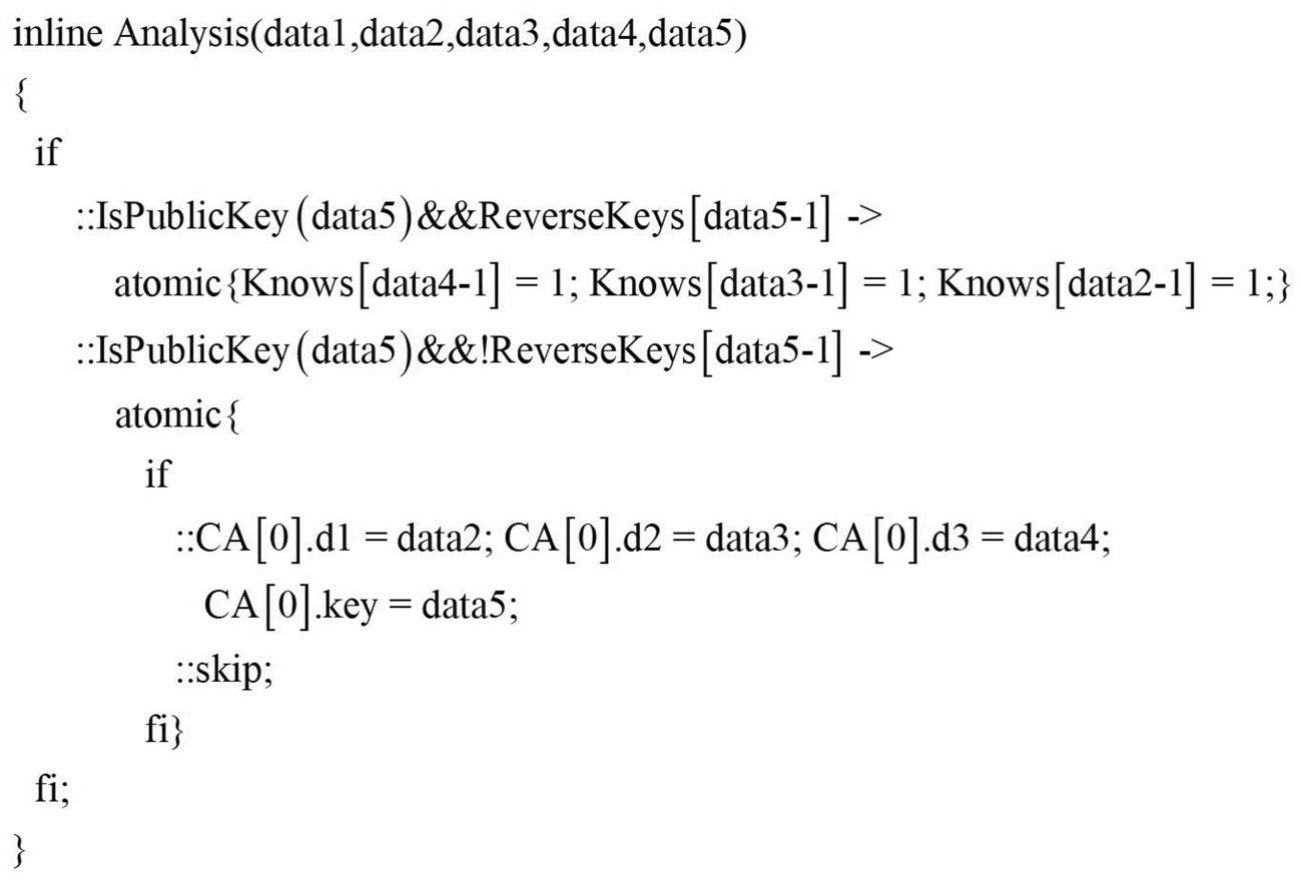
Analysis function.

In this paper, the main loop of the intruder’s process includes two concurrent sentences. According to the principle of grammatical reordering, the intruder’s receiving sentence is placed before the sending sentence to ensure that the intruder can obtain more knowledge. The first concurrent sentence (lines 5-6) of the intruder’s process is the first half of the BS behavior pattern $\mathrm{{(Intercept}} \rightarrow \mathrm{{Analysis}})$, which means that the intruder intercepts message and learns unknown knowledge. The behavior of intercepting the message is implemented by Algorithm 1, and the Analysis function is Algorithm 2 is implemented. As shown in Fig.10, the analysis function includes two statements. The first statement is used to determine whether the intruder can learn the unknown atomic message and expand it to the atomic knowledge set; The second sentence is used to expand the encrypted components that the intruder cannot deconstruct to the component knowledge library.

**Figure 11:**
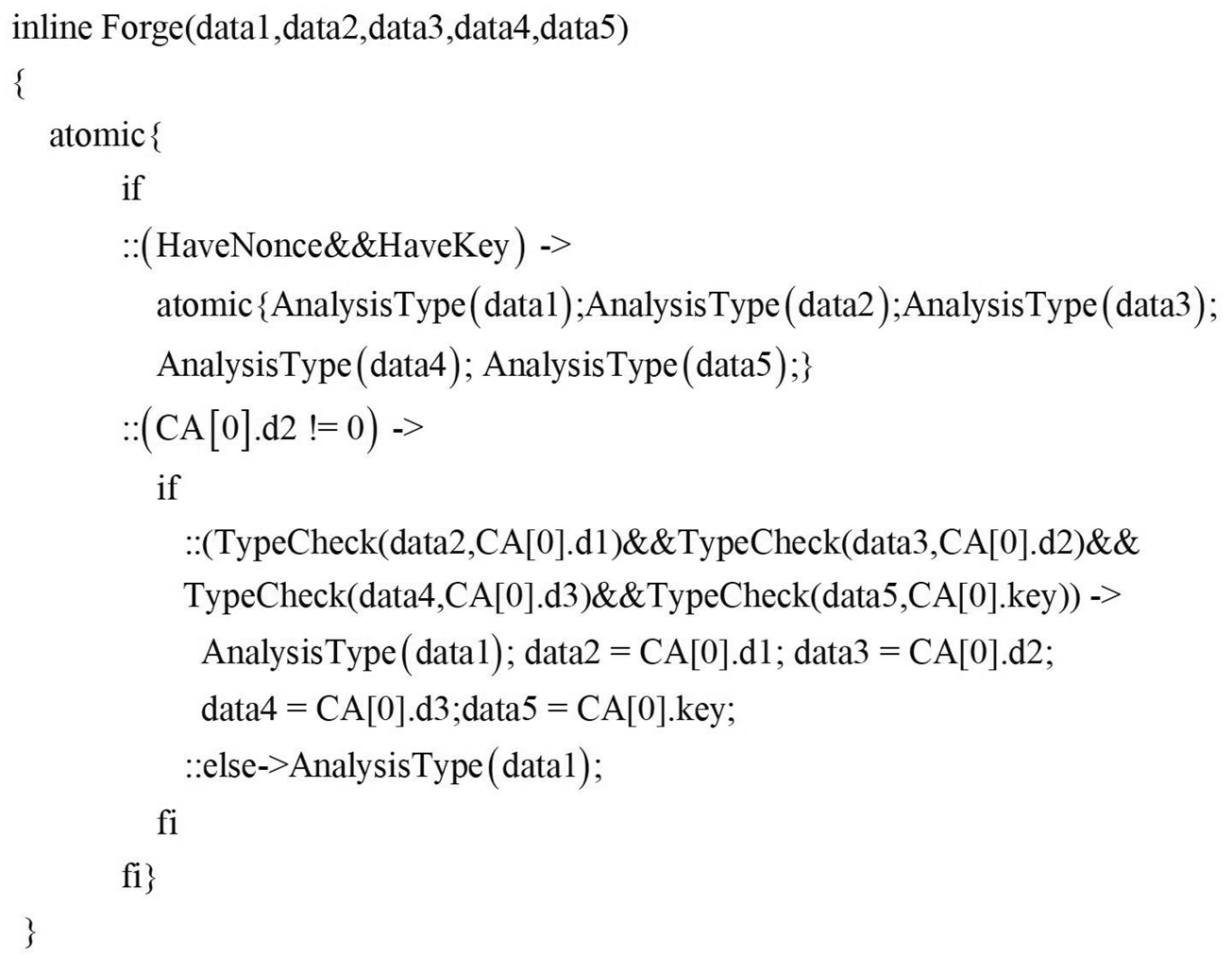
Forge function.

The second concurrent sentence of the intruder’s process is the second half of the BS behavior pattern $(\mathrm{{Forge}} \rightarrow \mathrm{{Send}})$, which means that the intruder forges and sends message. The forge behavior is implemented by Algorithm 3, and the send behavior is implemented by Algorithm 3. According to Algorithm 3, the specific implementation of the forged message function Forge in this paper is shown in Fig.11.

**Figure 12:**
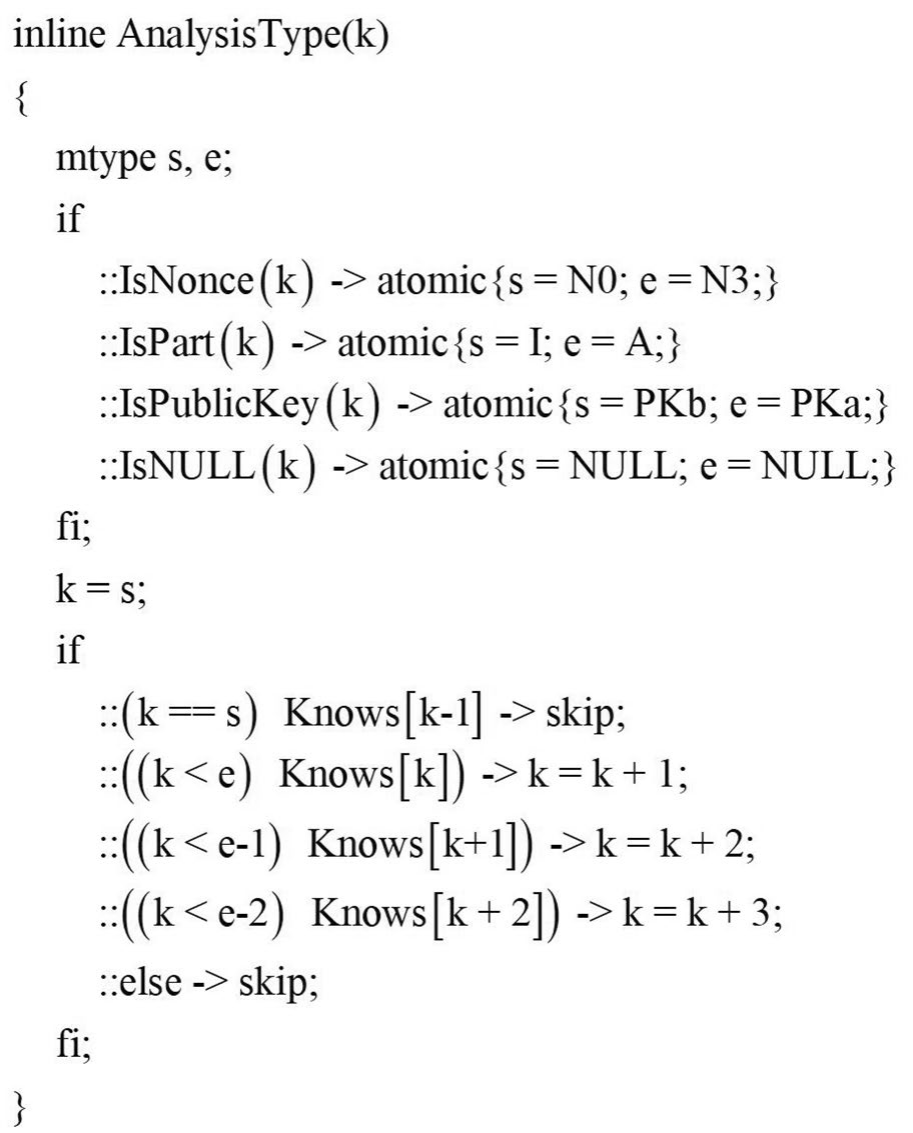
AnalysisType function.

Where HaveNonce and HaveKey are the macros to determine whether the intruder already has the random number and key, If the condition is passed, Intruder can choose to use his own atomic knowledge set to forge the message. That is, field detection is performed through the AnalysisType() function to forge elements that meet the current field type specification. Here, based on the principle of gradual enhancement of the intruder’s ability, we first deprive the attacker of the ability to generate random numbers to detect the protocol. The specific implementation is shown in Fig.12, among them, IsNonce, IsPart, IsNULL, etc. are all macros for judging the field type.

If the content of the component library is not zero, the intruder can also judge which messages can be forged and conform to the current message template $\varpi$ by the combination of atomic knowledge recognition and component recognition. The macro TypeCheck in Fig.11 is used to detect whether the message type of the current field matches the corresponding position type in the component library.

### Security properties verification

Next, we will verify the security properties that the composition protocol must meet. In that the NSB protocol is a flawed protocol, we verify the secrecy and authentication of the NSL protocol under the compositional operating environment of the NSB and NSL protocols.

- Secrecy
  Secrecy means that the secret cannot be known by entities other than the communicating entity during the process of protocol communication. In this paper, the secrecy verification is achieved through the secrecy assertion claim(r, secret, x). According to the previous definition of secrecy, the specific implementation is as follows.
  $$\begin{aligned} \begin{array}{*{20}{l}} {\# \mathrm{{define Seclnv}}\left( \mathrm{{x}} \right) \mathrm{{ (Knows}}\left[ {\mathrm{{x - 1}}} \right] ! = 1)}\\ {\mathrm{{inline Sec(x,other)}}\{ }\\ {\;\;\;\;\left( {!\mathrm{{Seclnv}}\left( \mathrm{{x}} \right) \& \& \mathrm{{other ! = I}}} \right) - > \mathrm{{ assert}}\left( {\mathrm{{Seclnv}}\left( \mathrm{{x}} \right) } \right) ;}\\ \} \end{array} \end{aligned}$$
  The secrecy assertion event is integrated in the protocol specification of each agent, as shown in Fig.7, lines 13-14. The agent of the protocol executes the secrecy assertion event according to the claim rule in Fig.4. When the intruder learns the secret x and the intruder is not the intended correspondent of the agent of the protocol, the assertion is violated.
- Authentication
  According to the formal definition of authentication in the security property part, we define four byte-type global variables to verify the authentication of the composition protocol through security assertion events. The global variables that express the properties of authentication are defined as follows.
  $$\begin{aligned} \begin{array}{*{20}{l}} {\mathrm{{byte IniRunningAB = 0;}}}\\ {\mathrm{{byte IniCommitAB = 0;}}}\\ {\mathrm{{byte ResRunningAB = 0;}}}\\ {\mathrm{{byte ResCommitAB = 0;}}} \end{array} \end{aligned}$$
  The macro $\mathrm{{IniRunning}}$ is the transparent event ${\varepsilon _ \downarrow }$ expanded in this article, used to update the value of the global variable $\mathrm{{IniRunningAB}}$. The other variables are also updated with corresponding macros.
  $$\begin{aligned} \begin{array}{*{20}{l}} {\# \mathrm{{define IniRunning}}\left( {\mathrm{{x,y}}} \right) \mathrm{{ if}}\backslash }\\ {{:}{:}\left( {\mathrm{{x = = A y = = B}}} \right) -> \mathrm{{ IniRunningAB}} + + ;\backslash }\\ {{:}{:}\mathrm{{else }} - > \mathrm{{ skip}};\backslash }\\ \begin{array}{l} \mathrm{{fi}}\\ \# \mathrm{{define IniCommit(x,y) if}}\backslash \\ ... \end{array} \end{array} \end{aligned}$$
  $\mathrm{{IniRunningAB + 1}}$ indicates that initiator A participated in a session with responder B in the protocol, and $\mathrm{{ResCommitAB + 1}}$ indicates that responder B submitted a session with initiator A. Therefore, the authentication of A to B is realized as the value of the variable $\mathrm{{IniRunningAB}}$ is greater than or equal to the value of the variable $\mathrm{{ResCommitAB}}$, and the reverse authentication properties are consistent. This can solve the problem of implementing authentication properties in multiple rounds of sessions and composition protocol verification. The authentication assertion events integrated on the agent of the protocol are as follows.
  $$\begin{aligned}\begin{array}{*{20}{l}} \begin{array}{l} \# \mathrm{{define AuthBtoA}}\backslash \\ \mathrm{{(ResRunningAB> = IniCommitAB)}} \end{array}\\ \begin{array}{l} \# \mathrm{{define AuthAtoB}}\backslash \\ \mathrm{{(IniRunningAB> = ResCommitAB)}} \end{array}\\ {!\mathrm{{AuthBtoA -> assert}}\left( {\mathrm{{AuthBtoA}}} \right) ;}\\ {!\mathrm{{AuthAtoB - > assert}}\left( {\mathrm{{AuthAtoB}}} \right) ;} \end{array} \end{aligned}$$
  Among the two protocols in this paper, NSB is a flawed protocol. We can compose it with the correct protocol NSL to find a composition protocol attack that violates the properties of NSL. In addition, the implementation method of authentication property in this paper can also be used to verify the injective consistency^33^. For example, as the initiator, A thinks that he and responder B have completed the execution of the agreement twice, but B actually only ran once. It would violate the authentication properties of AuthAtoB.

## Experiment Results

The experiment environment of this paper is: intel i5 CPU, 64 bits Linux, 4G RAM, SPIN V6.5.1. To prove the versatility and efficiency of the intruder model in this paper, first use the modeling method in this paper to formally verify the NSPK protocol, RPC protocol, TMN protocol, and Ban-Yahalom protocol in a single protocol environment. The experimental results are shown in Table 1.

**Table 1:** Experimental results of independent protocol analysis using the state reduction method in this paper.

Protocol	Method	Number of state	Number of transitions
NSPK protocol	This paper	76	90
	Ref.^25^	291592	524929
	Ref.^30^	826	1138
RPC protocol	This paper	21	21
TMN protocol	This paper	312	321
Ban-Yahalom protocol	This paper	2498	6808

Note: The data in Table 1 refers to a computer with an Intel(R) Core(TM) i5-4210H (2.90 GHz) and 8G memory configuration. On a virtual machine running 64-bit Linux and 4RAM, spin 6.5.1 Execute the built model and obtain the experimental results in the depth-first search mode (NSPK protocol, RPC protocol, and TMN protocol are the results of the default search depth of 10000, and the Ban-Yahalom protocol is the experimental result of the limited search depth of 90).

The experiment results show that the improved protocol model in this paper can effectively verify the security protocol and improve the efficiency of protocol verification.

**Figure 13:**
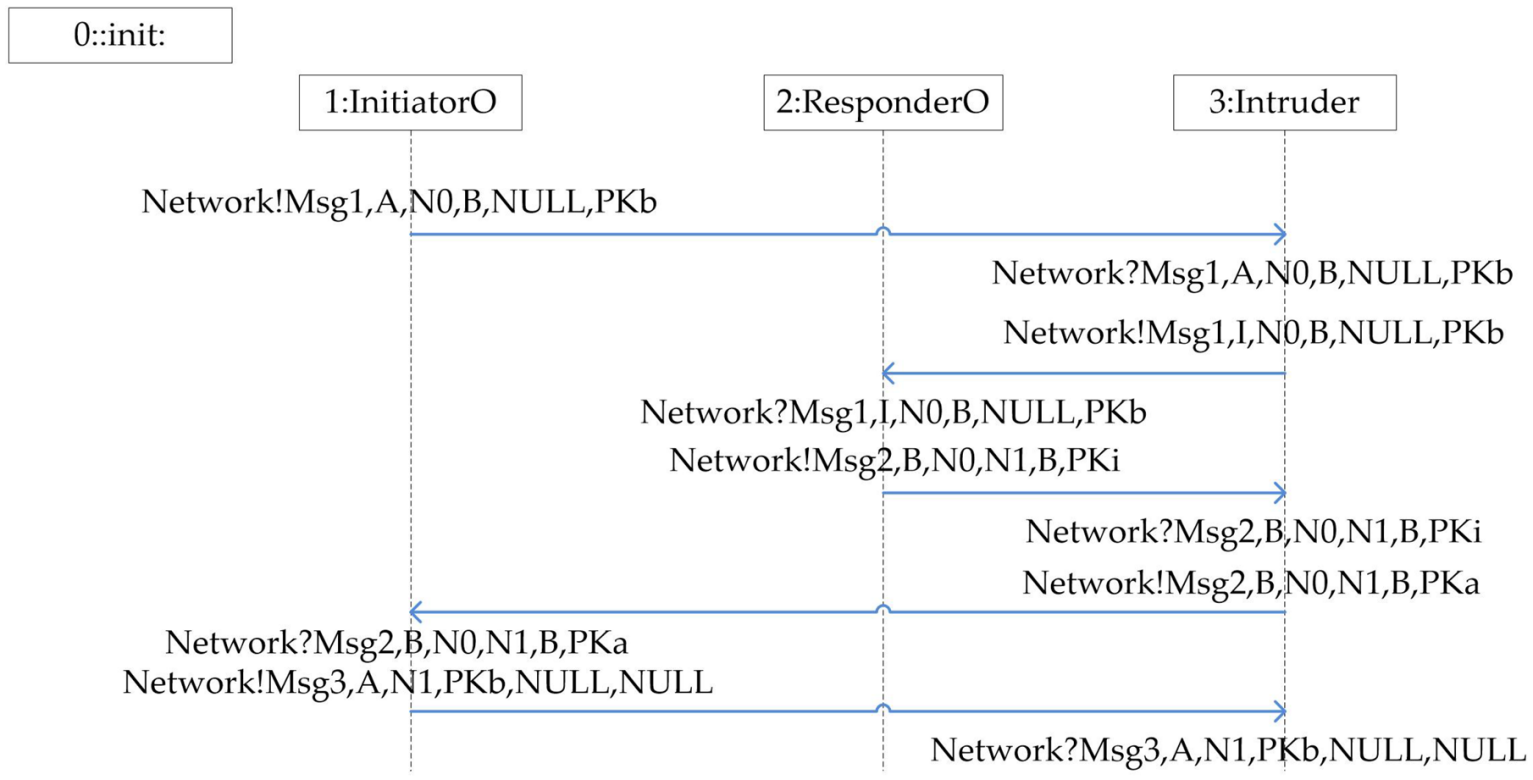
NSB breaks secrecy and authentication attack path.

Then through the previous method to model the composition protocol of the NSL and NSB protocols, a known attack that violates the secrecy and authentication in the composition protocol is successfully discovered in the depth-first search mode. The following shows the experiment results of a single protocol, as shown in Figs.13.

**Figure 14:**
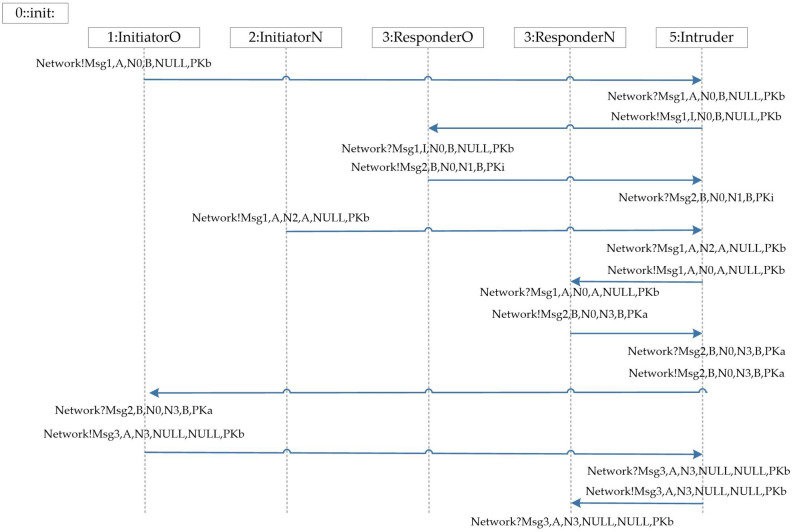
The NSL and NSB composition protocol violates the secrecy and authentication attack path.

From the experiment results of a single protocol, it can be seen that NSB violates secrecy and authentication, while NSL satisfies secrecy and authentication. Next, the results of the NSL and NSB protocol combination are shown, as shown in Table 2, Fig.14.

**Table 2:** The results of verifying the security of NSB and NSL composition protocol using the method in this paper.

Protocol	Method	Number of state	Number of transitions
NSB and NSL	This paper	24401	2901
	Ref.^25^	–	-

Note: The data in Table 1 refers to a computer with an Intel(R) Core(TM) i5-4210H (2.90 GHz) and 8G memory configuration. On a virtual machine running 64-bit Linux and 4RAM, spin 6.5.1 Execute the built model and obtain the experimental results in the depth-first search mode. - Indicates state explosion, no visible data.

The attack sequence of the NSL and NSB composition protocol attack is summarized as follows: 1) First, agent A executes the old protocol NSB to send message 1 to B, which is intercepted by the intruder.

$$\begin{aligned} \mathrm{{NSB:A}} \rightarrow \mathrm{{I}}\left( \mathrm{{B}} \right) \mathrm{{:A,}}\left\{ {\mathrm{{N0,B}}} \right\} \mathrm{{PKb}} \end{aligned}$$

2) Intruder I replayed the last message to responder B of the NSB protocol in his own capacity.

$$\begin{aligned} \mathrm{{NSB:I}} \rightarrow \mathrm{{B:I,}}\left\{ {\mathrm{{N0,B}}} \right\} \mathrm{{PKb}} \end{aligned}$$

3) Responder B continues to send message 2 to I in accordance with the protocol specifications of the old NSB protocol.

$$\begin{aligned} \mathrm{{NSB:B}} \rightarrow \mathrm{{I:B,}}\left\{ {\mathrm{{N0,N1,B}}} \right\} \mathrm{{PKi}} \end{aligned}$$

4) At this time, the intruder I pretends to be A, and uses the random number N0 obtained from the NSB protocol to initiate the new protocol NSL protocol to the agent B.

$$\begin{aligned} \mathrm{{NSL:I}}\left( \mathrm{{A}} \right) \rightarrow \mathrm{{B:A,}}\left\{ {\mathrm{{N0,A}}} \right\} \mathrm{{PKb}} \end{aligned}$$

5) After the agent B receives the session request of the NSL protocol, it sends message 2 to the initiator A it thinks according to the rules of the new protocol.

$$\begin{aligned} \mathrm{{NSL:B}} \rightarrow \mathrm{{A:B,}}\left\{ {\mathrm{{N0,N3,B}}} \right\} \mathrm{{PKa}} \end{aligned}$$

6) For the last two sentences of the NSB and NSL protocols are similar, the agent A sends message 3 to the agent B according to the rules of the NSB protocol after receiving the message, and the agent B receives the message. At this time, agent B believes that it has completed the authentication of the NSL protocol with the agent A. In fact, it has completed the authentication of the NSB protocol with the agent A. The authentication assertion of the NSL protocol violates, and the N0 that should be kept secret in the NSL protocol is also attacked by the intruder. It is learned that the secrecy security assertion of the NSL protocol N0 is violated.

$$\begin{aligned} \mathrm{{NSL:A}} \rightarrow \mathrm{{B:A,}}\left\{ {\mathrm{{N3}}} \right\} \mathrm{{PKb}} \end{aligned}$$

It can be seen from the attack sequence that this composition protocol attack has mutual communication between two protocol agents, and it is also likely to occur in actual protocol update scenarios. The experimental results prove that the method in this paper is effective for formal analysis and verification of the composition protocol.

## Summary and Future Work

The SPIN tool is used for the first time to verify the security of the composition protocol, and the general method is introduced in this paper, which provides directions and ideas for SPIN-based composition protocol formal verification. In this method, we detailed formal descript protocol operation semantics and related properties in the context of composition protocol applicable to SPIN. In addition, we also proposed methods for field detection and component recognition, which improve the efficiency of the model and can be better applied to the composition protocol verification environment. Since we only considered the parallel combination of multiple protocols, the analysis and verification of multiple protocols under sequential combination will be studied in the next step, and SPIN tools will be used to verify and discover more composition protocol attacks to prove that the method in this paper is general and efficient. At the same time, we will continue to optimize the intruder model in this paper, improve the versatility of the model, and develop an automatic detection system for composition protocol attacks based on SPIN.

## Data Availability

All data generated or analyzed during this study are included in this published article.
